# Ethical challenges of digital health technologies: *Aadhaar*, India

**DOI:** 10.2471/BLT.19.237123

**Published:** 2019-01-17

**Authors:** Vijayaprasad Gopichandran, Parasuraman Ganeshkumar, Sambit Dash, Aarthy Ramasamy

**Affiliations:** a Employees State Insurance Corporation Medical College and Post Graduate Institute of Medical Sciences and Research, Chennai, India.; b Indian Council of Medical Research, National Institute of Epidemiology, Chennai, India.; c Department of Biochemistry, Melaka-Manipal Medical College, Manipal Academy of Higher Education, Karnataka, India.; d Madras Diabetes Research Foundation, No. 4, Conran Smith Road, Gopalapuram, Chennai, 600086, India.

## Abstract

**Problem:**

The proliferation of information and communication technologies in India has enabled the emergence of health-related digital applications, from which important ethical issues arise.

**Approach:**

The *Aadhaar* identification system provides each resident in India with a 12-digit unique identification number, linked to demographic and biometric data. Identification by *Aadhaar* in welfare programmes has the important advantage of ensuring targeted benefits reach the intended recipients.

**Local setting:**

Some of the major issues faced by the public health sector in India are inadequate funding and inefficient utilization of the funds allocated. The enhancement of currently available digital health records will greatly increase the efficiency of the health care services.

**Relevant changes:**

The *Aadhaar* identification system has been linked to several health programmes since 2013. Success was achieved in a programme encouraging pregnant women to undergo delivery at a health facility, as use of *Aadhaar* number ensured that cash incentives reached the correct recipient. However, interruptions in the treatment of patients with tuberculosis and acquired immunodeficiency syndrome have been reported in other health programmes, due to patients fearing a breach of their confidentiality.

**Lessons learnt:**

Although the proposed merging of the *Aadhaar* identification system with digital health care records could enable greater efficiency in monitoring public health and welfare programmes, important ethical issues of privacy and data ownership and use must be considered. In joining the digital revolution, low- and middle-income countries must also develop strict legal regulation to protect data and avoid information technology companies exploiting such databases for profit.

## Résumé

**Problème:**

La prolifération des technologies de l'information et de la communication en Inde a favorisé l'émergence d'applications numériques liées à la santé, soulevant des questions éthiques.

**Approche:**

Le système d'identification *Aadhaar* fournit à chaque personne résidant en Inde un numéro d'identification unique à 12 chiffres relié aux données démographiques et biométriques. L'identification par *Aadhaar* dans le cadre de programmes de protection sociale représente un avantage décisif : elle permet de s'assurer que les allocations ciblées sont effectivement attribuées aux bénéficiaires adéquats.

**Environnement local:**

Le secteur de la santé publique en Inde rencontre plusieurs problèmes majeurs, dont un financement insuffisant et une utilisation inefficace des fonds alloués. L'optimisation des dossiers médicaux numériques actuellement disponibles va considérablement améliorer les performances des services de soins de santé.

**Changements significatifs:**

Le système d'identification *Aadhaar* a été couplé à de nombreux programmes de santé depuis 2013. Grâce à lui, un programme visant à encourager les femmes enceintes à accoucher dans un établissement médical a été couronné de succès : l'utilisation du numéro *Aadhaar* a servi à vérifier que les primes d'incitation en espèces étaient versées aux bons bénéficiaires. Cependant, des interruptions de traitement chez des patients souffrant de tuberculose et du syndrome d'immunodéficience acquise ont été observées dans d'autres programmes de santé ; les patients craignaient une violation de la confidentialité.

**Leçons tirées:**

Même si la proposition de fusion entre le système d'identification *Aadhaar* et les dossiers médicaux numériques pourrait accroître l'efficacité de la surveillance exercée sur les programmes de santé et de protection sociale, d'importants enjeux éthiques doivent être pris en compte en matière de confidentialité, mais aussi de propriété et d'utilisation des données. S'ils souhaitent opérer une révolution numérique, les pays à faible et moyen revenu doivent également adopter des dispositions légales strictes afin de protéger les données et d'éviter que des entreprises actives dans le secteur des technologies de l'information n'exploitent de telles bases de données à des fins lucratives.

## Resumen

**Situación:**

La proliferación de las tecnologías de la información y la comunicación en la India ha permitido el desarrollo de aplicaciones digitales relacionadas con la salud, de las que se derivan importantes cuestiones éticas.

**Enfoque:**

El sistema de identificación de *Aadhaar* proporciona a cada residente en la India un número de identificación único de 12 dígitos, vinculado a datos demográficos y biométricos. La identificación de *Aadhaar* en los programas de bienestar social tiene la importante ventaja de garantizar que las prestaciones específicas lleguen a los destinatarios previstos.

**Marco regional:**

Algunos de los principales problemas a los que se enfrenta el sector de la salud pública en la India son la financiación inadecuada y la utilización ineficiente de los fondos asignados. La mejora de los registros digitales de salud disponibles actualmente aumentará en gran medida la eficiencia de los servicios de atención sanitaria.

**Cambios importantes:**

El sistema de identificación de *Aadhaar* se ha vinculado a varios programas sanitarios desde 2013. Se logró el éxito en un programa que alentaba a las mujeres embarazadas a dar a luz en un centro sanitario, ya que el uso del número de *Aadhaar* garantizaba que los incentivos en efectivo llegaran al destinatario correcto. Sin embargo, en otros programas sanitarios se ha informado de interrupciones en el tratamiento de pacientes con tuberculosis y síndrome de inmunodeficiencia adquirida, por temor de los pacientes a que se vulnere su confidencialidad.

**Lecciones aprendidas:**

Aunque la fusión propuesta del sistema de identificación de *Aadhaar* con los registros digitales de atención sanitaria podría permitir una mayor eficacia en la supervisión de los programas sanitarios y de bienestar social, deben considerarse importantes cuestiones éticas de privacidad y propiedad y uso de los datos. Al sumarse a la revolución digital, los países de ingresos bajos y medios también deben elaborar una reglamentación jurídica estricta para proteger los datos y evitar que las empresas de tecnología de la información exploten esas bases de datos con fines de lucro.

## ملخص

**المشكلة:**

أدى انتشار تقنيات المعلومات والاتصالات في الهند إلى تمكين ظهور تطبيقات رقمية متعلقة بالصحة، والتي تنشأ عنها مشكلات أخلاقية هامة.

**الأسلوب:**

إن نظام *Aadhaar * لتعريف الهوية يزود كل مقيم في الهند برقم تعريف فريد مكون من 12 رقماً، وهو يرتبط بالبيانات السكانية والإحصائية الحيوية. يتميز نظام Aadhaar لتعريف الهوية في برامج الرعاية الاجتماعية بميزة هامة، وهي ضمان وصول المزايا المستهدفة إلى المستفيدين المستهدفين.

**المواقع المحلية:**

إن التمويل غير الكاف والاستخدام غير الفعال للأموال المخصصة، هي بعض المشكلات الرئيسية التي يواجهها قطاع الصحة العامة في الهند. إن تحسين السجلات الصحية الرقمية المتاحة حالياً، سوف يزيد بشكل كبير من كفاءة خدمات الرعاية الصحية.

**التغيّرات ذات الصلة:**

تم ربط نظام *Aadhaar * لتعريف الهوية بالعديد من البرامج الصحية منذ عام 2013. وتم تحقيق النجاح في برنامج يشجع النساء الحوامل على الخضوع للولادة في منشأة صحية، حيث أن استخدام رقم *Aadhaar * يضمن وصول الحوافز النقدية إلى المستفيد الصحيح. ومع ذلك، فقد تم الإبلاغ عن انقطاعات في علاج المرضى الذين يعانون من مرض السل ومتلازمة نقص المناعة المكتسب في برامج صحية أخرى، وذلك بسبب خوف المرضى من انتهاك خصوصياتهم.

**الدروس المستفادة:**

على الرغم من أن الدمج المقترح لنظام Aadhaar لتعريف الهوية، مع سجلات الرعاية الصحية الرقمية، يمكن أن يتيح قدراً أكبر من الكفاءة في مراقبة برامج الصحة العامة والرفاهية، إلا أنه يجب مراعاة القضايا الأخلاقية الهامة المتعلقة بالخصوصية وملكية البيانات واستخدامها. عند مواكبة الثورة الرقمية، فإن الدول ذات الدخل المنخفض والدخل المتوسط يجب عليها أيضاً وضع لوائح قانونية صارمة لحماية البيانات وتجنب قيام شركات تقنية المعلومات باستغلال قواعد البيانات هذه بغرض تحقيق الربح.

## 摘要

**问题:**

印度信息资源的急剧增加和通信技术的不断发展促使与医疗相关的数字应用出现，由此引发了重要的伦理问题。

**方法:**

*Aadhaar* 身份识别系统为每位印度居民提供一个独一无二的 12 位身份证明编号，该编号与人口统计和生物识别数据相关联。*Aadhaar* 身份识别在福利计划中的应用可以发挥其重要优势，可确保有针对性地向预定受益者提供福利。

**当地状况:**

印度公共卫生部门面临的一些主要问题是资金不足和分配资金的使用效率低下。现有数字医疗记录的增加将大大提高医疗服务的效率。

**相关变化:**

自 2013 年以来，*Aadhaar* 身份识别系统已与多个医疗计划挂钩。一项鼓励孕妇在医疗机构分娩的计划取得了成功，因为使用 *Aadhaar* 编号可以确保将现金奖励正确地发给受益者。然而，据其他医疗计划报告称，由于患者担心违反其保密性，在结核病和获得性免疫缺陷综合征（艾滋病）患者的治疗方面出现了中断。

**经验教训:**

虽然提议将 *Aadhaar* 身份识别系统与数字医疗记录合并可以提高监测公共卫生和福利计划的效率，但隐私、数据所有权和数据的使用方面的重要伦理问题也必须予以重视。在参与数字化革命的进程中，中低收入国家还必须制定严格的法律法规保护数据，避免信息技术公司利用这些数据库谋取利益。

## Резюме

**Проблема:**

Развитие информационно-коммуникационных технологий в Индии привело к появлению цифровых приложений в сфере здравоохранения, использование которых повлекло возникновение важных этических проблем.

**Подход:**

Система идентификации *Aadhaar* присваивает каждому жителю Индии уникальный 12-значный идентификационный номер, привязанный к его демографическим и биометрическим данным. Идентификация с помощью системы *Aadhaar* в программах социального обеспечения имеет важное преимущество: она гарантирует, что целевые льготы попадут именно к тем получателям, которые в них нуждаются.

**Местные условия:**

Одной из основных проблем в секторе общественного здравоохранения в Индии является недостаточное финансирование, а также неэффективное использование выделенных средств. Совершенствование системы имеющихся к настоящему времени цифровых медицинских записей значительно повысит эффективность услуг в сфере здравоохранения.

**Осуществленные перемены:**

Начиная с 2013 года система идентификации *Aadhaar* привязана к нескольким программам здравоохранения. Это позволило успешно провести программу поощрения беременных к родам в больничных условиях, поскольку использование номера *Aadhaar* гарантировало, что денежные льготы попадут к получателям. Однако в других программах охраны здоровья сообщалось о том, что больные туберкулезом и синдромом приобретенного иммунодефицита прерывали лечение по причине опасений нарушения конфиденциальности.

**Выводы:**

Несмотря на то что предложенное слияние системы идентификации *Aadhaar* с системой цифровой медицинской документации может увеличить эффективность мониторинга программ в сфере общественного здравоохранения и социального обеспечения, следует учитывать важные этические проблемы принадлежности данных и обеспечения их конфиденциальности. Присоединяясь к цифровой революции, страны с низким и средним уровнями дохода должны также разрабатывать жесткие нормы законодательства для защиты данных, препятствующие их использованию компаниями, работающими в сфере информационных технологий, ради собственной выгоды.

## Introduction

Low- and middle-income countries have experienced a rapid and massive influx of digital and mobile technologies and their applications. This information and communication revolution has brought great benefits for the practice of public health in these countries. Several studies have shown evidence of effective digital applications in health including electronic health records, digital epidemiology and mobile health applications.^1^ With these massive strides in digital health, important ethical issues related to the collection, storage, use and dissemination of patients’ digital health information arise. 

The *Aadhaar* (Hindi, meaning foundation or base) system of India provides each citizen with a unique identification number, linked to demographic and biometric information about the individual. The National Health Mission established the health management information system across various states of the country, where everyone is also provided with a unique health identification number. Linking this unique health identification number with the *Aadhaar* system has been proposed; merging the digital identification system with digital health care records could enable greater efficiency in investigating public health, conducting relevant research, ensuring the accuracy of health-related information and monitoring welfare programmes.^2^ However, the availability of big data in health care, particularly in low- and middle-income countries, introduces unique challenges. We identify and discuss such challenges in this article.

## Local setting

The delivery of health care in India is characterized by a vast public health infrastructure, which is categorized into primary, secondary and tertiary care services, delivered by the government-funded health system and a private health sector. The public health system is funded by tax revenues and state-sponsored health insurance schemes, such as *Ayushman Bharat*, and the private health system is largely financed out of pocket and, to a small extent, by private health insurance. In recent years, digital health records have penetrated the public as well as private health systems; most public health data are now recorded in digital format. One of the major issues faced by the public health sector in India is inadequate funding and inefficient utilization of the funds allocated. The enhancement of currently available digital health records will greatly increase the efficiency of planning and budgeting for the health care services.

## Approach

Under the Unique Identification Authority of India, established under the Ministry of Electronics and Information Technology of the Government of India, the *Aadhaar* system provides a 12-digit unique identification number to every Indian resident who chooses to enrol as per the provision of the *Aadhaar* (Targeted Delivery of Financial and Other Subsidies, Benefits and Services) Act, 2016.^3^ The *Aadhaar* identification system records demographic data such as name, address, date of birth and sex, but also biometric data including ten fingerprints, two iris scans and a facial photograph.^4^

Identification by *Aadhaar* number yields several important advantages in development and welfare programmes in the country. Targeted interventions, such as subsidies, cash benefits and incentives provided by the state can reach the intended beneficiaries without pilferage or loss. The *Aadhaar* system has been increasingly linked to bank accounts, income tax accounts, mobile phone numbers, and social welfare programmes, such as disability and elderly pension schemes.

## Relevant changes

Since 2013, *Aadhaar* has also been linked to several health-related schemes.^2^ For example, the Maternal and Child Tracking System is an electronic database that records information about pregnant women in India, including details of antenatal care, delivery and child-related data up to immunization. A conditional cash transfer programme (*Janani Suraksha Yojna*) encourages women to undergo delivery in a health facility by providing a direct benefit transfer; combining this cash transfer programme with the Aadhar identification system ensures that the correct person receives the benefit. 

Other national health programmes, such as the Revised National Tuberculosis Control Program and the National AIDS Control Program, have recently started linking their databases with *Aadhaar*.^2^ The intended benefit of linking the patient databases to the *Aadhaar* number is to track the treatment of patients and ensure that any interruptions to treatment schedules or non-compliance is identified and acted upon. However, interruptions in the treatment of patients with tuberculosis were reported due to pressures to link their treatment registration numbers with their *Aadhaar* identification number.^5^ There have also been several reports of HIV-positive patients and patients with AIDS who were undergoing antiretroviral therapy but, fearing a breach of their privacy by allowing their *Aadhaar* number to be included in the AIDS treatment programme documents, they preferred to drop out of the treatment programme.^6^

## Lessons learnt

The summary of main lessons learnt is presented in Box 1.

Box 1 **Summary of main lessons learnt**

- Use of the *Aadhaar* identification number in health programmes in India has met with mixed success due to patients’ fears of loss of privacy.
- The proposed merging of the *Aadhaar* identification system with other digital health databases in India could enable greater efficiency in monitoring public health, but important ethical issues of data use and protection must be considered.
- Low- and middle-income countries must invest in developing strict legal regulations to protect data and avoid its exploitation for profit.

Linking sensitive private health information with the *Aadhaar* identification system introduces issues of potential breaches of privacy, data ownership and use, and the autonomy of individuals whose data have become available for analysis. Unlike the United Kingdom of Great Britain and Northern Ireland and the United States of America, who have strong data ownership and protection laws, India has the evolving Electronic Health Record Standards of 2016, which does not have the force of law. Data are owned by the state in a sense of stewardship in the United Kingdom and by the individual in the USA. The Indian Electronic Health Record Standards stipulate that data are owned by individuals, but the state is the custodian of these data. This provision implies that any use of electronic health records requires the authorization of the individual. The exceptions to this rule are situations of emergency and epidemics, in which case the state can collect information without permission. Where data are shared without consent, this is only in a completely anonymized form.^7^

The *Aadhaar* identification system has been the subject of several litigations in various courts in India. In a ruling in 2013, the Supreme Court of India clearly ruled that registering for an *Aadhaar* identification number is voluntary and that nobody should be denied benefits and services because they do not have an *Aadhaar* number. Despite this ruling, several national health programmes continue to enforce *Aadhaar* registration in subtle ways. For example, although including an individual’s *Aadhaar* number is not compulsory in many public health programmes, some health workers continue to insist on this. If patients refuse, the health worker can delay or even prevent the receipt of benefits. In 2017, the Supreme Court ruled that the Right to Privacy is fundamental. This ruling reignited the various debates on the constitutional position of the *Aadhaar* system and whether it is an infringement of the privacy of individuals. In 2018, the Supreme Court upheld the constitutional position of *Aadhaar*, but ruled again that it is not mandatory.^8^ However, in the absence of strong regulation of electronic health records in low- and middle-income countries, linking sensitive health information to the *Aadhaar* system results in the privacy of patients being compromised.

Other major ethical concerns regarding the linking of digital health data and *Aadhaar* identification data are the safety and protection of the combined database. Many low- and middle-income countries do not yet have the required data protection laws; even if such laws are present, implementation can be difficult to monitor. Leaks of the *Aadhaar* data have been reported; a private telecommunication company in India collected the *Aadhaar* numbers of many of its subscribers and published them on the internet. Over 200 government websites were also found to be inadvertently displaying *Aadhaar* data of individuals.^9^ Although these websites have been taken down, such data can remain in the digital world indefinitely and continue to compromise the privacy of an individual.

Linking digital health data with the *Aadhaar* identification system also facilitates the exploitation of the data for profit. Some technologists who were initially involved in the establishment of the *Aadhaar* system are now working in private for-profit companies. Although security measures are in place to prevent this conflict of interest, there are several loopholes in these regulations.^10^ When such profit-motivated, digitally empowered multinational companies gain access to an identification database in low- and middle-income countries, this disproportionately affects the poor and vulnerable. Effective security systems and strong legislation are required to ensure that private companies do not profit from accessing such databases. In this context, the Ministry of Electronics and Information Technology tabled the Draft Personal Data Protection Bill, 2018. When passed, this bill will set up a national Data Protection Authority to supervise and regulate those who collect data, ensuring the autonomy of individuals.^11^

Low- and middle-income countries are joining the digital revolution, making big data available for potential health applications. However, weak health data protection laws and evolving data protection capabilities in these countries leave the population vulnerable to serious ethical consequences, such as breaches in privacy and loss of autonomy. In examining the merging of *Aadhaar* identification data with digital health data in India, we have demonstrated the need for strong health data protection laws to preserve and protect the fundamental human right: that of privacy.
